# All-in-One digital microfluidics pipeline for proteomic sample preparation and analysis†

**DOI:** 10.1039/d3sc00560g

**Published:** 2023-02-22

**Authors:** Jiaxi Peng, Calvin Chan, Shuailong Zhang, Alexandros A. Sklavounos, Maxwell E. Olson, Erica Y. Scott, Yechen Hu, Vigneshwar Rajesh, Bingyu B. Li, M. Dean Chamberlain, Shen Zhang, Hui Peng, Aaron R. Wheeler

**Affiliations:** a Department of Chemistry, University of Toronto 80 St. George Street Toronto ON M5S 3H6 Canada aaron.wheeler@utoronto.ca +1-416-946-3865 +1-416-946-3866; b Donnelly Centre for Cellular and Biomolecular Research, University of Toronto 160 College Street Toronto ON M5S 3E1 Canada; c Institute of Biomedical Engineering, University of Toronto 164 College Street Toronto ON M5S 3G9 Canada; d Lunenfeld-Tanenbaum Research Institute, Mount Sinai Hospital 600 University Avenue Toronto ON M5G 1X5 Canada; e School of Environment, University of Toronto 33 Willcocks Street Toronto ON M5S 3E8 Canada; f School of Mechatronical Engineering, Beijing Institute of Technology Beijing 100081 China; g Beijing Advanced Innovation Center for Intelligent Robots and Systems, Beijing Institute of Technology Beijing 100081 China; h Saskatchewan Cancer Agency, University of Saskatchewan 107 Wiggins Road Saskatoon SK S7N 5E5 Canada; i Clinical Research Center for Reproduction and Genetics in Hunan Province, Reproductive and Genetic Hospital of CITIC-XIANGYA Changsha Hunan 410000 China

## Abstract

Highly sensitive and reproducible analysis of samples containing low amounts of protein is restricted by sample loss and the introduction of contaminants during processing. Here, we report an All-in-One digital microfluidic (DMF) pipeline for proteomic sample reduction, alkylation, digestion, isotopic labeling and analysis. The system features end-to-end automation, with integrated thermal control for digestion, optimized droplet additives for sample manipulation and analysis, and an automated interface to liquid chromatography with tandem mass spectrometry (HPLC-MS/MS). Dimethyl labeling was integrated into the pipeline to allow for relative quantification of the trace samples at the nanogram level, and the new pipeline was applied to evaluating cancer cell lines and cancer tissue samples. Several known proteins (including HSP90AB1, HSPB1, LDHA, ENO1, PGK1, KRT18, and AKR1C2) and pathways were observed between model breast cancer cell lines related to hormone response, cell metabolism, and cell morphology. Furthermore, differentially quantified proteins (such as PGS2, UGDH, ASPN, LUM, COEA1, and PRELP) were found in comparisons of healthy and cancer breast tissues, suggesting potential utility of the All-in-One pipeline for the emerging application of proteomic cancer sub-typing. In sum, the All-in-One pipeline represents a powerful new tool for automated proteome processing and analysis, with the potential to be useful for evaluating mass-limited samples for a wide range of applications.

## Abstract 2

The All-in-One digital microfluidic pipeline allows for end-to-end automation of processing and analysis of mass-limited proteomic samples.

## Graphical abstract

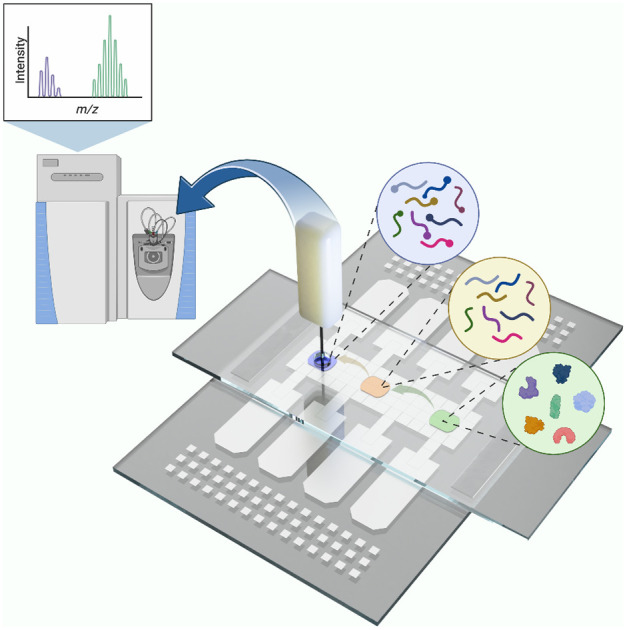

## 1 Introduction

Mass spectrometry (MS)-based proteome profiling has emerged as a powerful, unbiased technology for identification and quantitation of the set of expressed proteins found in complex biological samples.^1,2^ The most common approach is “bottom up” profiling, in which proteins are digested into constitutive peptides prior to analysis by HPLC-MS/MS and database searching to identify the proteins that correlate with the constitutive peptides. State-of-the-art analysis techniques can identify such peptides when present in zeptomole quantities.^3,4^

There are many exciting current trends in bottom-up proteome profiling for applications in biomedicine. One important trend focuses on pushing the boundary for how many proteins can be identified from tiny samples, such as the lysate generated from a single mammalian cell. As recently as 3–5 years ago, the record for such studies was a few hundred proteins per cell.^5–7^ More recently, methods that integrate ion mobility spectroscopy (IMS) in-line with traditional MS approaches have pushed this record to thousands of proteins per cell.^8–11^ Another trend in bottom-up proteome profiling for applications in biomedicine is the development of strategies in which samples are differentially labeled with mass-tags, to allow for semi-quantitative comparisons between them.^12–14^ These techniques, which are increasingly being applied to clinical cancer typing,^15,16^ typically prioritize reproducible detection over the absolute number of proteins identified. The current report falls squarely in this category of application.

Whether a particular study is focused on label-free identification of large numbers of proteins per cell, or label-enabled semi-quantitative comparisons between samples, all bottom-up proteome profiling techniques suffer from inevitable sample loss that occurs during the extensive sample processing that is required prior to analysis.^17,18^ Specifically, the number of steps, the number of reaction vessels, and the process of transferring the sample from a reaction system to the high performance liquid chromatography-mass spectrometry (HPLC-MS) system are all known to increase the probability of non-specific adsorption, sample loss and contamination.^19,20^ Numerous sample preparation technologies for mass-limited proteomic samples have been developed to address this challenge, including SP3,^21^ SODA,^22^ nanoPOTS,^5^ and on-column techniques.^23^ While these are important developments, the operation requirements are typically quite high, such as access to robotic nanoliter liquid handling or complex capillary and column connections, which are not accessible to all analysts (even in well-equipped labs).

One potential solution to the challenges indicated above is automated processing by digital microfluidics (DMF), a miniaturized fluid-handling technique that manipulates samples and reagents as picoliter-to microliter-sized droplets, typically by application of electrodynamic forces on an array of electrodes, for automated sample processing and reactions.^24^ DMF has been viewed for many years as being a useful tool for processing samples upstream of analysis by mass spectrometry and proteomics.^25,26^ The smaller volumes in DMF systems provide some advantages for reactions (including rapid heat transfer into and out of the system), and the enclosed system and automated control of DMF reduces the probability of experimental variation and contamination that can be introduced during manual operations. DMF has previously been used in proteomics research to extract analytes from single cells,^7^ to extract proteins from mixtures by precipitation,^27^ immunodepletion,^28^ and immunoenrichment,^29^ as well as to implement protein digestion and related processes,^30–36^ and mass-tag labeling.^37^ These techniques represent important steps forward for the field, but none are fully integrated and automated – in each of these examples, there are still many manual transfers required, which takes away from some of the potential advantages of the technology.

Here, we describe the first fully integrated and automated system for digital microfluidic proteome sample handling and analysis, to facilitate what we call the “All-in-One” DMF pipeline for proteomic sample processing. In developing this system, we encountered numerous challenges, including: (a) complexities in ensuring temperature control for digestion and other steps, (b) incompatibility of DMF droplet constituents with HPLC-MS analysis, and (c) efficient sampling between DMF and HPLC-MS. Here we describe our solutions to these challenges, realizing a fully integrated process and analysis system. Importantly, the system was applied to the differential analysis of human breast cancer tissue samples, demonstrating its potential use for clinical cancer sub-typing. We propose that the All-in-One pipeline may be appropriate for a wide range of applications going forward.

## 2 Results and discussion

### 2.1 Droplet control on DMF

**Fig. 1:**
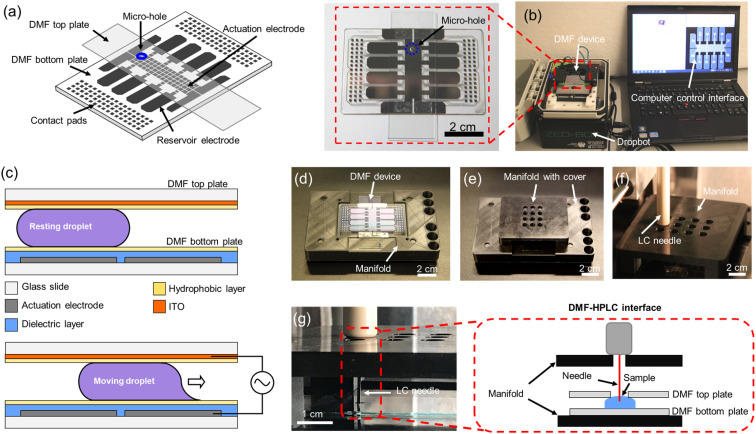
All-in-One digital microfluidic (DMF) pipeline for proteomic sample processing and analysis. (a) Cartoon of the DMF device used here, including a bottom plate and a top plate featuring a sampling micro-hole (blue). (b) Photograph of a DMF device interfaced with the open-source DropBot control system and computer running the open-source MicroDrop program. Inset: closeup photograph of a DMF device. (c) Schematics of DMF device (side-view) illustrating the components of the DMF device, including glass substrates (white), actuation electrodes on the bottom plate (grey), the dielectric layer on the bottom plate (blue), the hydrophobic layers (yellow), and the indium tin oxide (ITO) counter-electrode on the top plate (orange). When no electric potential is applied (top), the droplet is immobile. When an electric potential is applied to a particular driving electrode (bottom), the droplet moves onto the electrode. Photographs of customized DMF-autosampler manifold without (d) and with (e) the custom cover. (f) Photograph of the top cover of the manifold bearing sampling array holes that support the autosampler injector needle. (g) Photograph and schematic (inset) illustrating the sampling process in the DMF-HPLC interface.

The primary goal of this project was to develop a sample-to-answer pipeline for bottom-up proteomic sample processing with mass-tag labeling to allow for semi-quantitative comparisons between clinical samples. As a first step towards this goal, a DMF system was developed to integrate digestion, labeling, and loading processes. A standard DMF device is shown in Fig. 1a with a DropBot^38^ liquid processing unit (version 3.0) running MicroDrop software (version 2.31.1) to control droplet movement (Fig. 1b). As shown in Fig. 1c, droplets in this system are sandwiched between two plates and are manipulated by applying AC potentials between driving electrodes on the bottom plate and the counter-electrode on the top plate. Micro-holes were fabricated in the top plates to allow for droplet extraction after sample preparation (Fig. 1a and b inset). In this system, DMF acts not only as a protein reactor, but also as a liquid handler and an interface to HPLC-MS to minimize sample loss and contamination.

### 2.2 Customized interface between DMF and LC

A number of techniques have been developed to facilitate direct infusion of processed analytes from a DMF device into a mass spectrometer, including the powerful “on-the-fly” technique.^26^ These methods are useful for many applications, but bottom-up proteome profiling typically requires high-resolution chemical separations prior to analysis by MS, meaning that the All-in-One pipeline must interface with an HPLC (upstream of the MS). In most DMF/proteomic analyses described previously,^7,28–37^ processed samples were manually extracted from the device and then transferred to the autosampler/HPLC (risking sample loss and cross-contamination). The one exception is a previous report^20^ of a direct interface between DMF and an HPLC autosampler. Here, we introduce an improved DMF-autosampler interface (Fig. 1d and e) and describe its use in an All-in-One (sample-to-answer) protein processing pipeline.

The new DMF-autosampler interface relies on a custom 3D printed manifold (see STL file included as ESI†) that comprises (i) a base to hold the DMF device, (ii) four wells for the alignment of the injector, (iii) six slots for conventional injection vials, and (iv) a cover with sampling array holes to guide and support the injector needle. Compared with the previous interface,^20^ which aligned the autosampler to arrays of vials, the new interface was designed to align with 96-well-plates that are commonly used in modern HPLC instrumentation, including the EASY-nLC 1200 ultra-high-pressure system used here. The base of the manifold fits the shape of the DMF bottom plate, which precisely positions the DMF device to avoid damaging the injector needle of the autosampler. The cover with sampling array holes is detachable, which facilitates straightforward insertion and removal of the DMF device. In all, the manifold has the same dimensions as a standard multiwell plate, which should provide compatibility with a wide range of instruments in use today.

The sample loading process involves contact between the injector needle and the autosampler manifold, extension of the capillary needle, and finally suction of the liquid from DMF into the sample loop of the autosampler (Fig. 1f and g). Autosamplers are designed to work with conical-bottomed multiwell plates to facilitate complete collection (or “loading”) of samples into the needle. The DMF interface has a flat/planar bottom, which motivated us to evaluate whether there were differences in loading efficiencies between the two systems. Identical volumes of BSA digest standard (prepared manually) were sampled in triplicate using both formats, and the relative loading efficiency was determined by evaluating the average MaxQuant^39^ protein intensity of a BSA digest sampled from the DMF system relative to the same mixture sampled from wells in conical bottomed well plate. As shown in Fig. S1,† the relative loading efficiencies were quite similar: 98.9% for the DMF system relative to the well plate, and indeed, in visual inspections, there does not seem to be liquid left behind on the DMF devices after loading, likely facilitated by the hydrophobic DMF device surfaces (with aqueous contact angles ≥115°). In sum, the two systems appear to be roughly equivalent, noting a small increase in variance for the DMF system that might be improved in the future with additional engineering.

### 2.3 Optimization of surfactant concentration

Surfactants are important in bottom-up proteome profiling, as the addition of an appropriate surfactant can improve recovery by reducing non-specific protein adsorption to surfaces, as well as aiding in enhancing digestion efficiency.^40,41^ But proteomics researchers must be careful with this strategy, as the wrong surfactant can severely suppress the MS signal during HPLC-MS identification. This challenge has led to considerable innovation in the development of surfactants that are designed specifically for MS compatibility, which have become especially popular for the analysis of low-volume samples.^5,40–43^ For methods relying on digital microfluidics, there is an additional challenge: some surfactants have the right combination of surface tension and viscosity to enable DMF droplet movement (and importantly, dispensing without “tailing”), while others do not.^44–46^ Unfortunately, the surfactants that are often used in DMF (including pluronics and tetronics), interfere with peptide detection of MS. Therefore, the choice of surfactant and its concentration for a proteomic workflow on DMF is critical. In previous (independent) reports, we^7^ and Tholey and co-workers^37^ have found success in using maltoside derivatives that are known to be useful for proteomics [including *n*-dodecyl-β-d-maltoside (DDM) and 3-dodecyloxypropyl-1-β-d-maltopyranoside (DDOPM)] as additives for droplet manipulation on DMF devices. In our previous report,^7^ the optimum working concentration of DDM was not thoroughly investigated, and in fact, a high concentration/high viscosity/low velocity solution of 0.0125% wt/wt was used. Thus, a primary goal for our work here was to rationally assess and optimize the concentration of DDM for analysis.

**Fig. 2:**
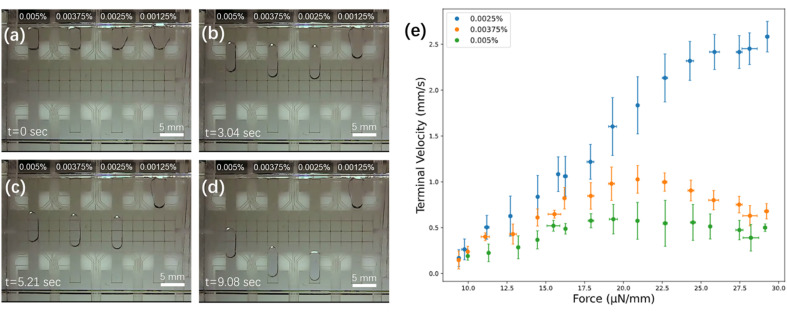
Effect of DDM concentration on droplet movement. Frames from Movie S1† illustrating the movement of aqueous droplets containing DDM (0.0025, 0.00375, and 0.005% wt/wt) after (a) 0 s (during the loading step), (b) 3.04 s, (c) 5.21 s, and (d) 9.08 s. (e) Force–velocity curves for the three highest concentrations tested (0.0025% – blue, 0.00375% – orange, 0.005% – green). Error bars are ± 1 st. dev. for *n* = 3 replicates per condition.

Force–velocity curves^47^ were generated for four concentrations of DDM (0.00125%, 0.0025%, 0.00375%, and 0.005%, wt/wt in water) to identify optimum working conditions. As illustrated in Fig. 2a–d and Movie S1,† the lowest concentration tested (0.00125%) was found to be challenging to load into the device, while the three higher concentrations (0.0025%, 0.00375%, and 0.005%) loaded easily and moved smoothly and reproducibly. Note that aqueous samples with low or no surfactant can be loaded manually into DMF devices by pipetting onto a bottom plate and then sandwiching them with a top plate, but in this work, it was desirable to identify conditions in which samples could be loaded automatically from reservoirs at the side of the device. Among the three solutions that loaded easily, the lowest concentration (0.0025%) moved the most rapidly and with largest saturation force (∼25 μN mm^−1^). This concentration, which is substantially lower than those reported previously,^7,37^ was used for all subsequent experiments, allowing for rapid and efficient DMF operations.

### 2.4 All-in-One pipeline

Bottom-up proteomic sample processing requires the execution of a long list of repetitive and iterative reaction-steps (including reduction, alkylation, and digestion), while maintaining careful control over temperature, pH, and light level. This complexity is exacerbated when mass-tag labeling is included, which requires that multiple replicates of each sample (exposed to different tags) be carried through the same set of steps in parallel. The DMF community has previously demonstrated the feasibility of on-chip proteomic reactions^30–36^ or mass tag labeling,^37^ but never both, and never before have either set of methods been combined with automated loading into HPLC-MS. Here, we set out to develop a method that does all of these things, which we dub the “All-in-One” DMF proteome analysis pipeline.

**Fig. 3:**
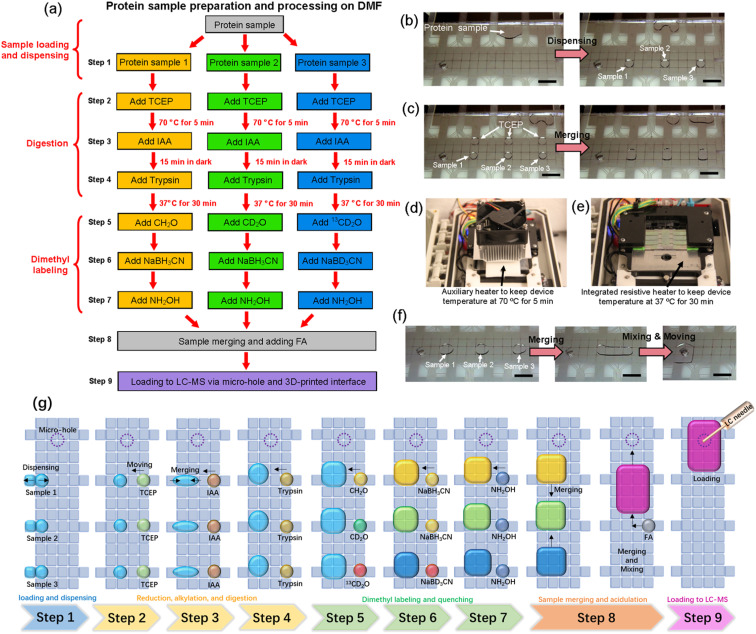
The All-in-One DMF proteomic pipeline. (a) Workflow illustrating the All-in-One pipeline that includes sample loading (step 1), reduction (step 2), alkylation (step 3), digestion (step 4), isotopic labeling and quenching (steps 5–7), processed sample pooling (step 8) and loading into the HPLC-MS (step 9). Samples labeled with light, medium, and heavy mass-tags are illustrated in orange, green, and blue, respectively. Photographs illustrating various steps in the pipeline, including (b) step 1, (c and d) step 2, (e) step 4, and (f) step 8. (g) Schematic illustrating the entire workflow.

The nine steps in the All-in-One pipeline are listed in Fig. 3a. Each step involves multiple repetitions of standard DMF operations, including dispensing (or metering) of reagents onto the device (Fig. 3b) and merging reagents together so that they react (Fig. 3c). The open-source DropBot^38^ control system used here was modified to include two automated heating units – an auxiliary heating unit (introduced here for the first time) was used to denature the sample at 70 °C during disulfide reduction by exposure to TCEP (Fig. 3d), and an integrated unit (reported previously^48,49^) was used to incubate the sample at 37 °C during enzymatic digestion by exposure to trypsin, after an alkylation reaction by exposure to IAA (Fig. 3e), both programmed by the user and controlled by pulse-width modulation (PWM). The two different heating units were found to be necessary because the auxiliary heating unit allowed for much faster heating and cooling to minimize evaporation. Temperature profiles generated during replicate experiments using the two systems are shown in Fig. S2.† Samples are then mass-tagged in multiple steps by exposure to isotopically labeled formaldehyde (CH_2_O, CD_2_O, and ^13^CD_2_O) and sodium cyanoborohydride (NaBH_3_CN and NaBD_3_CN), and then the reactions are quenched by exposure to hydroxylamine. Finally, samples are acidified by exposure to formic acid and loaded into the autosampler *via* the manifold described above (Fig. 1d–g). An advantage of including mass-tags in the procedure is that the samples bearing different labels can be pooled prior to analysis (Fig. 3f). In some experiments, the entire pooled sample was loaded into the autosampler, while in others, a portion of it was loaded, suggesting the possibility (in the future) for carrying part of the sample forward for additional processing prior to analysis. Finally, other labeling schemes might be used in the future; dimethyl labeling was selected here because it can be used with virtually any kind of sample.^13^ A schematic summarizing all of the steps for three samples is shown in Fig. 3g.

**Fig. 4:**
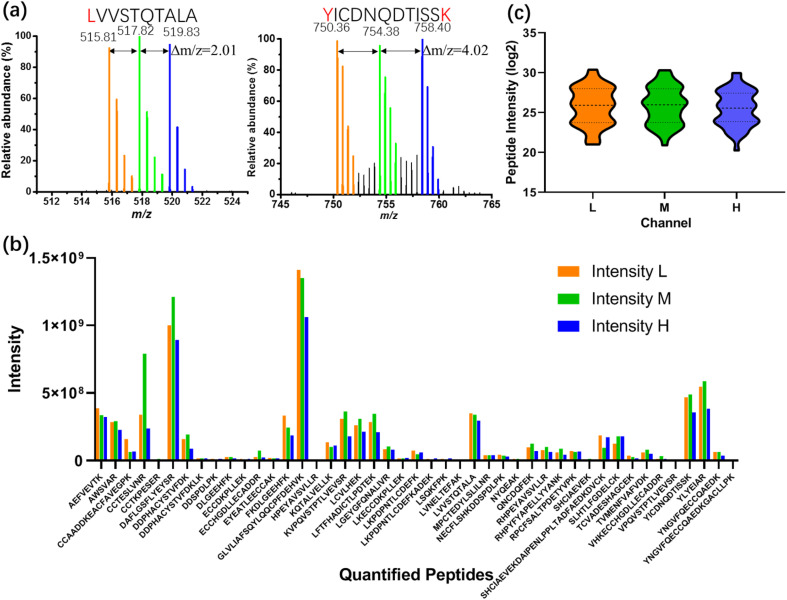
Dimethyl labeling of All-in-One pipeline-processed BSA samples on DMF (1 ng of BSA for each channel). (a) Extracted mass spectra of LVVSTQTALA (left) and YICDNQDTISSK (right) with light (orange), medium (green) and heavy (blue) label. (b) Intensity distributions of each of the 45 quantified peptides bearing light (L, orange), medium (M, green) and heavy (H, blue) labels. (c) Violin plot of distribution of quantified peptide intensity (log 2) from light (L, orange), medium (M, green) and heavy (H, blue) labeled channel.

As a first test of the All-in-One pipeline, a BSA standard was processed according to the scheme illustrated in Fig. 3a, labeling sub-samples with isotopic light, medium, or heavy dimethyl labels (nominally +28, +32, and +36 Da per labeled residue, respectively). Spectra from All-in-One pipeline-processed samples are shown in Fig. 4a. As expected, singly labeled peptides like LVVSTQTALA (labeled at the N-terminus) are separated by *m*/*z* differences of 2.01 (Fig. 4a-left), while doubly labeled peptides like YICDNQDTISSK (labeled at the N-terminus and at the lysine residue) have *m*/*z* differences of 4.02 (Fig. 4a-right). In all, 54 BSA peptides were identified, including 45 peptides with each of the labels (which were thus were amenable for quantitation) (Fig. 4b). This number of “quantified” analytes (that are identified in each sample and replicate tested) is critical for the type of comparative proteome profiling applications that motivated this work. The distribution of intensities of the quantified peptides between the different samples was similar (Fig. 4c), with a relatively low average coefficient of variation (CV) of 27.2% (Fig. S3†). More importantly, the intensities of more than 70% of the quantified peptides (32/45) had CVs between the tagged peptide intensities of less than 30%, a common threshold^50,51^ that is applied in mass-tag experiments for quantitation between samples.

As a control for the All-in-One DMF pipeline performance (Fig. 4 and S3†), the same amount of BSA standard was dispensed into tubes and reagents were added by pipette. From the samples processed in tubes, 49 peptides were identified in all, and 41 peptides were present in each of the sub-samples (and thus were amenable for quantitation) (Fig. S4a†). The variation of identified peptide intensity between and within the labeled groups (Fig. S4b†) was larger than that of the BSA processed by the All-in-One DMF pipeline. Specifically, the average CV of the intensities of the quantified peptides prepared in tubes was 75.6% (Fig. S5†), and less than 10% of the quantified peptides (4/41) had a CV smaller than 30%.

There are many potential explanations for the improved performance of the All-in-One DMF pipeline relative to manual processing in tubes. One possible explanation is the potential for improved digestion kinetics (arising from improved thermal transfer in the DMF system), resulting in fewer missed cleavages. To test this hypothesis, a mixture of 5 protein standards was digested on DMF and in tubes. As shown in Fig. S6,† the distribution of missed cleavages in identified peptides for each of proteins was similar for the two methods, suggesting that digestion kinetics were not a dominant factor in the improved performance of the DMF pipeline. With this in mind, we suspect that the difference in labeling reproducibility may be attributed to improved metering reliability and decreased numbers of different surfaces exposed to the sample (reducing risk of unpredictable non-specific adsorption) in the All-in-One DMF pipeline. More work will be required to know for sure, but regardless of the reason, the performance was acceptable for the goals described here, which led us to apply it to a proof-of-concept application, cancer cell-line profiling.

### 2.5 Application of All-in-One DMF pipeline to cancer cell line profiling

The ultimate goal of this work was to develop a technique useful for cancer sub-typing,^15,16^ an emerging technique in which isotopically labeled proteomes are compared to identify protein expression patterns that correlate with different forms and stages of metastatic cancer. As an intermediate step toward this goal, two breast cancer cell lines, MCF-7 and MDA-MB-231, were evaluated using the All-in-One pipeline. These samples were selected because they are known to have dramatic differences in cell invasion behavior, with MDA-MB-231 cells being more invasive into a collagen-I-rich matrix, a proxy for aggressive metastatic disease.^52^

**Fig. 5:**
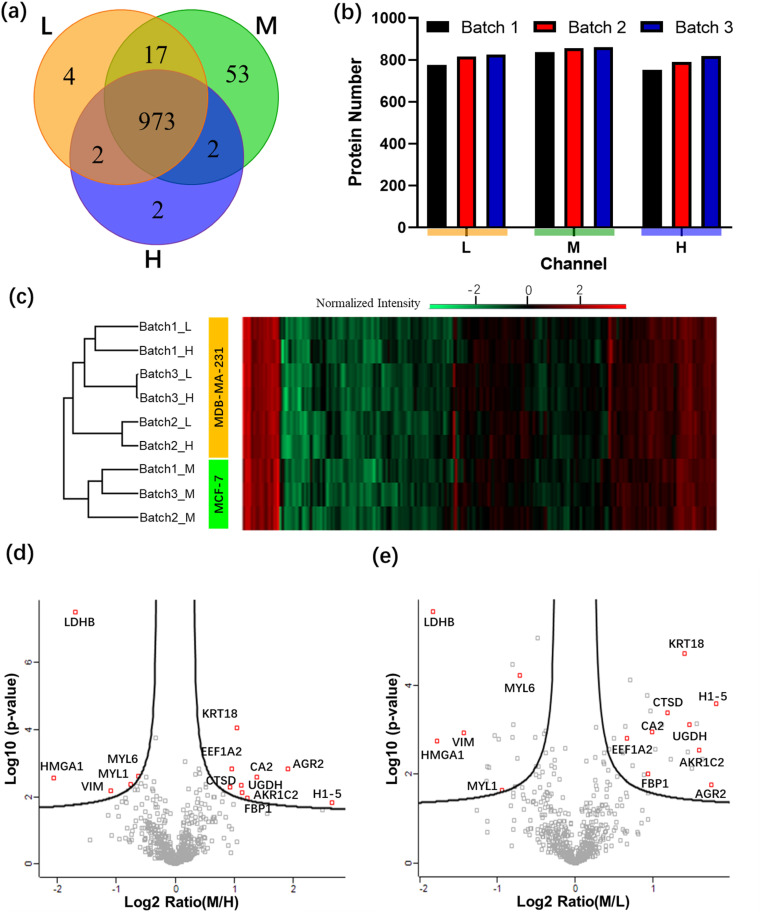
Dimethyl labeling of cell lysates using the All-in-One DMF pipeline. (a) Venn diagram of proteins identified from three batches of light (“L”, MDA-MB-231 cells), medium (“M”, MCF-7 cells), and heavy (“H”, MDA-MB-231 cells) channels. (b) Plot of the number of proteins identified in each channel from the three batches in the light (“L”) medium (“M”) or heavy (“H”) channels. (c) Heat map of the 597 quantified proteins (columns) found in each of the nine samples (rows), including three batches (1, 2, 3) of light (“L”), medium (“M”), and heavy (“H”) channels. Red, black, and green shades represent proteins with high, median, and low abundance, respectively. Volcano plots of all proteins identified in the proteome database for the M/H channels (d) and M/L channels (e). Proteins that were differentially expressed between MCF-7 and MDA-MB-231 in both volcano plots are highlighted with red boxes.

In these experiments, MCF-7 cells were labeled with medium (M) isotope, while MDA-MB-231 cells were split and labeled with light (L) or heavy (H) isotopes. Assigning two channels to the same cell line (MDA-MB-231) allows them act as controls for one another as well as to serve as a reference to compare against the experimental MCF-7 channel (a practice that is commonly used to confirm repeatability in mass-labeling experiments^12^). Three replicates (or “batches”) of labelled samples were evaluated using the All-in-One pipeline. The correlation coefficients between protein intensities from channels L and H in batches 1, 2, and 3 were 91.5, 95.4, and 97.3%, respectively, confirming the repeatability of the measurements. A total of 973 proteins were identified across the three channels tested (Fig. 5a). The medium channel had the most proteins uniquely identified, which meets our expectation given that the other two channels comprised the same cell line (MB-MDA-231). Specifically, as shown in Fig. 5b, the average number of proteins identified in the light and heavy channels (containing MDA-MB-231 cells) was 806 ± 27 and 788 ± 27 (average ± standard deviation), respectively, while the average number of proteins identified in medium channel (containing MCF-7 cells) was 851 ± 13. The relative standard deviation of the number of protein identifications in each channel was less than 5%, further demonstrating the reproducibility of the All-in-One DMF workflow.

Finally, a heat map of the 597 quantitated proteins (identified in all nine samples) is shown in Fig. 5c. As expected, the three medium channels are clustered separately from the light and heavy channels. Volcano plots comparing these 597 proteins found in channels M/H (Fig. 5d) and M/L (Fig. 5e) were then prepared. The redundancy of this type of analysis (evaluating multiple batches of control *vs.* experimental) allows for assessments of differential expression with high confidence.^12^

Armed with a robust and reproducible comparative dataset, attention was turned to codifying the specific differences in protein expression between the two cell lines. From previous studies, we expected to observe three main distinctions: (i) MDA-MB-231 cells do not respond to hormones, whereas MCF-7 cells may;^53^ (ii) metabolic strategies differ between the cell types (MCF-7 cells prefer oxidative phosphorylation under normoxic conditions, whereas MDA-MB-231 cells prefer glycolysis^54,55^); and (iii) MCF-7 cells are more epithelial in morphology, while MDA-MB-123 cells are more mesenchymal.^56,57^

**Fig. 6:**
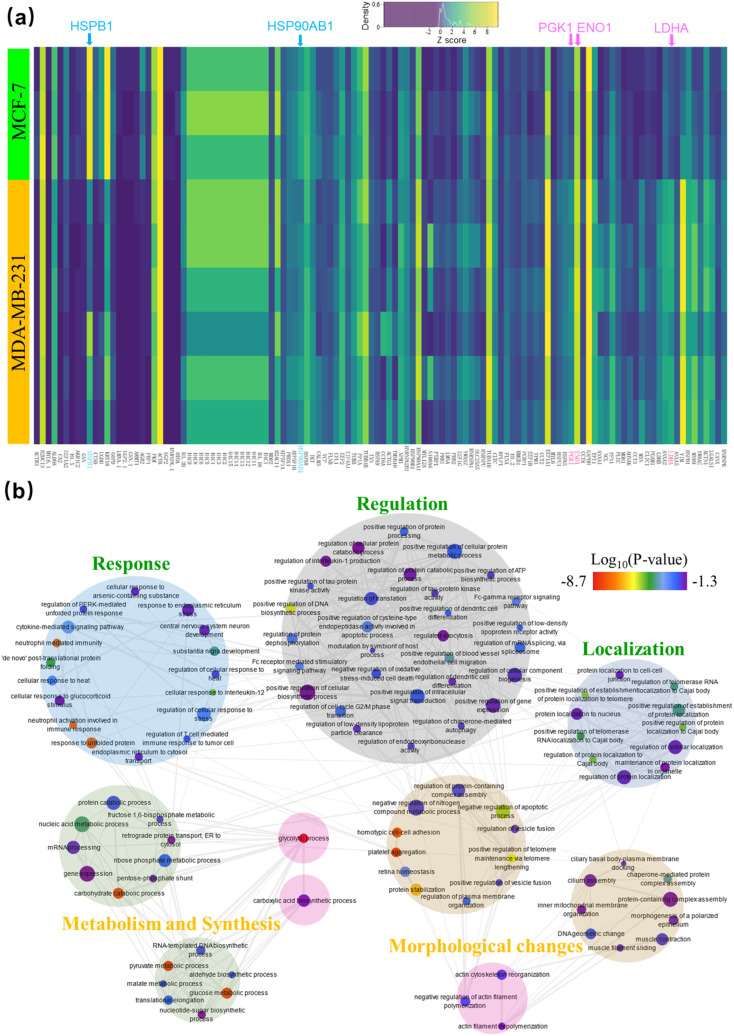
Analysis of the identified most variable proteins between MCF-7 and MDA-MB-231 determined using All-in-One DMF pipeline. (a) Heat map of 118 differentially expressed proteins (columns) found in the nine cell lysate samples (rows). Yellow, green, and dark grey shades represent proteins with high, median, and low abundance, respectively, and proteins associated with hormone pathways and glycolysis are highlighted in aqua and pink, respectively. (b) Graphical depiction of the biological processes represented by the 118 proteins from (a). Large circles define process groupings, markers define specific processes, marker color represents the log_10_(*p*-value) that reports the statistical confidence of protein function assignment (red-low, violet-high), marker size represents the frequency of the gene-ontology (GO) term in the underlying gene ontology annotation (GOA) database, and gray lines indicate relationships between the specific processes.

A heat map highlighting the expression of the 118 most variable proteins between the two cell lines is shown in Fig. 6a. Upon clustering of the normalized (*z*-score) protein intensities, distinct blocks of protein expression were found that confirmed our three key expectations. That is: (i) hormone pathway related proteins like HSP90AB1 and HSPB1/HSP27 (highlighted in aqua), are upregulated in MCF-7 cells;^58^ (ii) glycolysis-related proteins LDHA, ENO1 and PGK1 (highlighted in pink) are more abundant in MDA-MB-231 cells, and (iii) other proteins related to the epithelial morphology of MCF-7 (including KRT18 and AKR1C2) are among the most variable of the proteins.

The pathways represented by the 118 variably expressed proteins were then determined using Enrichr^59–61^(Fig. 6b). Pathways more active in MCF-7 cells included the estrogen signaling pathway (KEGG 2021 human, adjusted *p*-value = 1.84 × 10^−4^) and carboxylic acid biosynthetic process (GO Biological process, 0046394, adjusted *p*-value = 0.0289). The latter is related to the tricarboxylic acid (TCA) cycle, which has a prominent role in supplying the oxidative phosphorylation pathway.^62^ Likewise, pathways that were more active in MDA-MB-231 cells included those involved with glycolysis (KEGG 2021 human, adjusted *p*-value = 1.63 × 10^−10^) and actin myosin filament sliding (GO Biological Process, 0033275, adjusted *p*-value = 0.0195), the second of which is known to be more plentiful in the mesenchymal-like MDA-MB-231 cells.^56^ In sum, the differential pathway analysis supported the findings in the differential protein expression analysis, giving us confidence to move to evaluating clinical samples.

### 2.6 Analysis of human breast tissue using the All-in-One DMF pipeline

Building from encouraging results with breast cancer cell lines, we turned to applying the new technique to evaluate human breast cancer tissue samples, as a step toward an automated technique for cancer sub-typing.^15,16^ Three samples were acquired: healthy breast tissue (CP565563), a small breast cancer tumour that was either not metastatic or was collected early in the disease progress (CP531533), and a large, metastatic breast cancer tumour (CP629057). Upon evaluating the three samples with the All-in-One pipeline, 882 proteins were identified in total, and 436 proteins were quantified (found in all samples). Among this group, 31 proteins (Table S1,† CP531533) and 56 proteins (Table S2,† CP629057), respectively, were found to be differentially quantified for the two cancer samples relative to healthy tissue. Among this group (differentially quantified relative to healthy tissue), 15 proteins (higher quantities: PGS2, UGDH, ASPN, LUM, COEA1, PRELP; and lower quantitites: PDIA3, AMPL, PDIA4, B2MG, CALR, PLSL, 1A68, COR1A, D6RGG3) were commonly identified in both breast cancer tissue samples, and have been reported to be associated with the progression of breast cancer.^63–76^ The quantitative trends for these proteins were consistent in both breast cancer tissues, which further validated the reliability of identification and quantification by the All-in-One pipeline.

Intriguingly, 27 proteins were found to be differentially quantified in a comparison between the two cancer samples (Table S3†). For example, Decorin (PGS2) was found in higher quantities in the large/metastatic tumour relative to the small tumour. This protein, which is related to the regulation of estrogen^77^ is associated with tumor size and disease progression and outcome.^78^ Other proteins that were identified in the comparison between large/metatstatic tumour and small tumour were also flagged in the comparison between the invasive (MDA-MB-231)/non-invasive (MCF-7) cancer cell line study, including A0A1B0GW44 (CTSD) and K1C18 (KRT18). They are known promotors of aggressive tumour growth,^79,80^ which suggests that the All-in-One pipeline may be a valuable tool for correlating cellular function (*i.e.*, *in vitro* MDA-MB-231/MCF-7 invasion assays^52^) to *in vivo* progression of disease.

The data presented here demonstrate the potential utility of the All-in-One DMF pipeline for cancer sub-typing in clinical samples. These types of samples are by nature precious and mass-limited, and thus are particularly susceptible to the problem of analyte loss during processing. As described in the introduction, there are a number of emerging technologies^5,21–23^ that are being developed to address the problem of processing mass-limited samples with minimal exposure to surfaces. We propose that the All-in-One DMF pipeline is a useful addition to this list, bringing the combination of flexibility (with the capacity for real-time reconfigurability depending on the application) with end-to-end automation to bear. We anticipate that the proof-of-concept application described here will be the first of many to leverage these unique properties.

## 3 Conclusions

We have introduced an All-in-One DMF proteomic pipeline for digestion, isotopic labeling, and loading of miniaturized proteomic samples. Through the multiplexed control of DMF, up to three samples in the current system (potentially expandable to more in the future) can be processed simultaneously and then (after isotopic labeling) combined for analysis. By integrating localized heating and a direct-loading interface for HPLC-MS, user intervention is minimized, and the risk of sample loss and contamination is reduced. The pipeline has been applied to cancer sub-typing using model cell lines and clinical tissue samples. The system as described is appropriate for semiquantitative comparisons between different samples, and if in the future it can be combined with IMS, it may someday be appropriate to apply to cutting-edge single-cell protein identification experiments. In sum, we propose that the All-in-One DMF pipeline is a powerful new tool for automated proteome processing and analysis, with the potential to be useful for evaluating mass-limited samples for a wide range of applications.

## 4 Materials and methods

### 4.1 Reagents

Cell media reagents, acetonitrile (ACN), formic acid, and water (LC/MS-grade) were purchased from Thermo Fisher Scientific (Waltham, MA). Dimethyl labeling reagents, *n*-dodecyl-β-d-maltoside (DDM), tris(2-carboxyethyl)phosphine (TCEP), iodoacetamide (IAA), triethylammonium bicarbonate (TEAB), trypsin, and protein standards were obtained from Sigma-Aldrich (Oakville, CA). Lys-C was purchased from Promega (Madison, WI, USA).

### 4.2 DMF device fabrication and control

DMF devices comprising (i) bottom plates bearing 68 roughly square (2.2 × 2.2 mm) driving electrodes (where 60 of them formed a 4 row × 15 column electrode array) and 12 reservoir electrodes, and (ii) 25 × 75 mm top plates bearing a counter-electrode, were fabricated as described previously. Each top plate was modified by drilling a 2 mm dia. Micro-hole in the center of the piece (relative to the short axis) and approximately 23 mm from one edge (along the long axis) using a micro-drill press. Top plates and bottom plates were assembled with ∼190 μm-thick inter-plate spacers formed from two pieces of Scotch double-sided tape (3M), such that the access hole was positioned over row 2 and between columns 2–3 in the driving electrode array. This spacer defines “unit droplets” (with volume sufficient to cover a single driving electrode) of approx. 0.9 μL.

Devices were controlled by using a custom version of the open-source DropBot system^38^ (version 3.0, https://github.com/sci-bots/dropbot-v3).^48,49^ The DropBot and the built-in heater unit were controlled using a custom version of MicroDrop software (version 2.31.1, https://github.com/sci-bots/microdrop), while the auxiliary unit was controlled by a separate interface (in-house software, written in Python). Devices were interfaced to the DropBot system through a pogo-pin connector, and electrodes were actuated in pre-programmed steps which allowed droplet dispensing, moving, and mixing by applying (typically) 100 V_RMS_ as square waves at 10 kHz (parameters optimized as described below).

### 4.3 Auxiliary thermoelectric unit

An ESP32 Feather Board (Adafruit Industries, NY) was used to control a thermoelectric unit (TEC-30-32-127 Digi-Key, MN) through a VNH5019 motor driver (Pololu, NV). The thermoelectric unit was sandwiched between an aluminum plate (1.8 mm × 44 mm × 88 mm) (McMaster-Carr, IL) and a heatsink/fan unit (Cooler Master A73, Newegg, CA). The fan was connected to the VHN5019 motor driver, and was programmed to turn on during cooling cycles and off during heating cycles. Thermal paste (Thermal # 860, MG Chemicals) was applied on either surface of the thermoelectric unit to ensure good thermal conductivity with the aluminum plate and the heatsink. The ESP32 board was also connected to two Negative Temperature Coefficient (NTC) thermistors (10 kΩ, NTCG103JX103DTDS, TDK Corporation) which were placed in close proximity to the thermoelectric unit to provide real-time temperature feedback. Pieces of Delrin (6 mm × 46 mm × 20 mm) (McMaster-Carr, IL) were placed on either end of the heating board to protect and allow the user to move the board when it was hot. A magnet was affixed in one of the two Delrin pieces and was used to align and secure the board in place during operation.

Custom firmware was developed in C++ to allow the ESP32 board to communicate with and be controlled by the host computer over USB. The firmware implemented a proportional–integral–derivative (PID) algorithm for the fine control of the heating unit using the temperature readings from the thermistors. The PID algorithm was modified from previous versions^48,49^ to output both positive and negative values to facilitate heating and cooling respectively. The thermistor closest to the thermoelectric unit was used for feedback while the thermistor further away from the unit served as a monitor of the heat distribution over the heating block. The proportional term (*K*_p_), integral term (*K*_i_) and derivative term (*K*_d_) of the PID controller were tuned to 70 °C, and the maximum temperature was set by software to be 120 °C.

Custom software was developed in Python to communicate with the ESP32 board and control the heating unit. Using this software, a custom heat profile could be programmed where desired temperatures and heating profiles could be specified. The software also logged the temperature readings from both thermistors on the heating board.

### 4.4 DMF-HPLC manifold fabrication

A DMF-HPLC manifold was fabricated from poly-lactic acid filament (colorFabb B.V., Belfeld, NL) in two parts using an Ultimaker 2 3D printer (Ultimaker B.V., Utrecht, NL). Steel dowel pins (McMaster-Carr Supply Co, Elmhurst, IL) were used to align the top and bottom components and to position the device in the autosampler. The overall manifold dimensions are 144 mm L × 90 mm W × 33 mm H. An STL file for the design is included in the ESI.†

### 4.5 Surfactant concentration optimization

Aqueous solutions of TEAB (50 mM) were supplemented with DDM to final concentrations ranging from 0.00125% to 0.005% wt/wt. Using a modified version of a method described previously,^47^ force–velocity curves were generated to identify optimal working conditions. Briefly, double-unit droplets were dispensed onto the array and velocity was monitored as a function of driving force; each condition was repeated in triplicate. In subsequent experiments, 100 V_RMS_ driving voltage was used (corresponding to ∼30 μN mm^−1^).

### 4.6 Protein standards

The proteins evaluated included a BSA standard and a protein standard mixture (albumin, catalase, cadherin, haptoglobin, and serotransferrin). The former was prepared by dissolving 1 mg of BSA in 1 mL of DI water, and then diluted to 1 ng μL^−1^ in 0.0025% wt/wt DDM in 50 mM aqueous TEAB. The protein mixture solution was prepared by dissolving 1 mg of each of the proteins in 1 mL of DI water, and then diluting them to 1 or 10 ng μL^−1^ (of each protein) in 0.0025% wt/wt DDM in 50 mM aqueous TEAB.

### 4.7 All-in-One proteomic pipeline

Experiments were carried out in 9 steps, all at room temperature with reagents dissolved in 0.0025% wt/wt DDM (in 50 mM aqueous TEAB) unless specified otherwise. (1) Aliquots of protein sample and TCEP (25 mM) were loaded into separate reservoirs on the device. (2a) Three unit-droplets each of sample and TCEP solution (six droplets total) were dispensed onto the array, and each protein/TCEP droplet pair was merged and mixed. (2b) The auxiliary heater (as shown in Fig. 3d) was engaged at 70 °C for 5 min to denature the protein. (3) After cooling, an aliquot of IAA (60 mM) was loaded into a reservoir, and three unit-droplets of IAA were dispensed onto the array and merged and mixed with the reduced protein droplets. The system was incubated in the dark for 15 minutes. (4) An aliquot of trypsin (10 ng μL^−1^) was loaded into a reservoir, and three unit-droplets were dispensed onto the array and merged with the alkylated protein droplets at 37 °C (controlled by the built-in resistive heater, as shown in Fig. 3e) for 30 min. During this period, the droplets were continuously moved in a loop on the array. (5) Aliquots of isotope-labeled formaldehyde (0.2% w/v), comprising CH_2_O (light), CD_2_O (medium), or ^13^CD_2_O (heavy), were loaded into reservoirs, and one unit-droplet of each was dispensed and mixed with one of the sample droplets. (6) Aliquots (37.5 mM) of sodium cyanoborohydride (NaBH_3_CN) and sodium cyanoborodeuteride (NaBD_3_CN) were loaded into reservoirs, and unit droplets of the former were merged with the light- and medium-labeled samples, while a unit droplet of the latter was merged with the heavy-labeled sample. All samples were incubated for 30 min. (7) An aliquot of ammonium hydroxide (0.2% w/v) was loaded into a reservoir, and three unit-droplets were dispensed and merged with the sample droplets. (8) The three sample droplets were merged to form one pooled droplet, and then an aliquot of FA (10% w/v) was loaded into a reservoir, and one unit-droplet was dispensed and merged with the pooled droplet. (9) Finally, the merged droplet was moved to the micro-hole to be loaded onto the autosampler. Briefly, the device was interfaced with the custom manifold, which was loaded into the EASY-nLC 1200 ultra-high-pressure system, where the sample was aspirated into the sample loop at 3 μL min^−1^ until (in most cases) the pooled 18 μL droplet was injected. Protein mixtures evaluated in digestion efficiency experiments were processed in an abbreviated version of the pipeline comprising steps 1–4, after which samples were collected by pipette for manual injection into the autosampler. Manually processed samples were prepared using identical volumes and concentrations, but were manipulated by pipettes and microcentrifuge tubes, heated in a thermocycler (Bio-Rad), and finally transferred into conical-bottomed 96-well plates (Eppendorf), prior to loading into the EASY-nLC 1200.

### 4.8 Cell culture and cell samples

MCF-7 and MDA-MB-231 cells were grown in Dulbecco's Modified Eagle's Medium (DMEM) with 10% fetal bovine serum (FBS), penicillin (100 U ml^−1^), and streptomycin (100 μg ml^−1^) in T-25 culture flasks in a humidified incubator with 5% CO_2_ at 37 °C. Cells were trypsinized, washed with phosphate-buffered saline (PBS) three times (centrifuged at 300×*g*, 5 min), and then suspended in PBS at densities ranging from 50 000 to 100 000 cells per ml (determined *via* hemacytometer). 1 μL samples (about 50–100 cells) were aliquoted into tubes and stored at −80 °C until analysis. For analysis, sample tubes were thawed at room temperature and each sample was mixed (by pipette) with 1 μL of 25 mM TCEP in 0.005% wt/wt DDM. Each sample/TCEP mixture was pipetted onto a DMF device (three at a time), after which steps (2b)–(9) were carried out by DMF as above, except the digestion in step (4) was performed with a mixture of 5 ng μL^−1^ Lys-C and 5 ng μL^−1^ trypsin.

### 4.9 Human breast tissue samples

Human breast tissue lysates (Cat. No. CP565563, CP531533, and CP629057) were purchased from OriGene (Rockville, MD, USA). Each sample was diluted to 30 ng μL^−1^ total protein in 0.0025% wt/wt DDM in 50 mM aqueous TEAB. Aliquots (1 μL) of each diluted breast tissue lysate were processed according the standard nine-step All-in-One pipeline, except that the digestion in step (4) was performed with a mixture of 5 ng μL^−1^ Lys-C and 5 ng μL^−1^ trypsin, and the volume loaded into the autosampler in step (9) was approximately one third (∼6 μL) of the pooled droplet.

### 4.10 HPLC-MS/MS analysis

Experiments were performed on a Q Exactive HF-X mass spectrometer coupled with an EASY-nLC 1200 system (Thermo Fisher Scientific). Each sample was automatically loaded onto a C18 trap column (3 cm, 100 μm i.d., Polymicro Technologies) at a flow rate of 2 μL min^−1^. The sample was then eluted into a fused silica microcapillary column (12 cm, 100 μm i.d., Polymicro Technologies), packed in-house with 1.9 μm-diameter reversed phase C18 particles (ReproSil-Pur 120 Å, Dr Maisch GmbH). Mobile phase A (water with 0.1% formic acid, v/v) and mobile phase B (80/20/0.1% ACN/water/formic acid, v/v/v) were used to generate a linear gradient of 3–30% B for 90 min, followed by a linear increase from 30–45% B for 20 min, then a linear increase from 45–95% B for 1 min and maintaining at 95% B for 14 min. The flow rate was set to 300 nL min^−1^. A full mass scan collected by the Orbitrap mass analyzer was from *m*/*z* 375 to 1575 with a resolution of 120 000, while the automatic gain control (AGC) target was 5 × 10^5^ and maximum injection time was 50 ms. Precursor ions with charges of +2 to +6 were fragmented by using high energy collision with 27% normalized energy at a resolution of 60 000, AGC of 5 × 10^4^, and maximum injection time of 250 ms. Previously selected precursor ions were excluded from further sequencing for 20 s.

### 4.11 Proteomic data analysis

Raw data files were evaluated by MaxQuant (version 1.6.4.0), using methods similar to those described previously.^39^ Briefly, a human protein database downloaded from UniProt (release 2018_09) was used to search the MS/MS spectra, with methionine oxidation and N-terminal protein acetylation set as variable modifications and cysteine carbamidomethylation set as a fixed modification. Multiplicity was set to 3 with dimethLys0 and dimethNter0 as light labeling, dimethLys4 and dimethNter4 as medium labeling, and dimethLys8 and dimethNter8 as heavy labeling. Trypsin was set as specific proteolytic enzyme with maximum 2 missed cleavages for each peptide. The minimum peptide length was set as 6 amino acids and maximum peptide mass was 4600 Da, and the ‘re-quantify’ option was selected. Both peptides and proteins were filtered with a maximum FDR of 0.01. The default settings of MaxQuant were used for all parameters not mentioned, and the resulting protein intensities were used as proxies for the amount of each identified protein.

Nine samples were evaluated in cell lysate experiments: six MDA-MB-231 and three MCF-7; these data were subjected to two analyses. In analysis one, MaxQuant output files were evaluated using Perseus,^81^ to exclude proteins identified as decoys, potential contaminants, or those identified exclusively by one-site modification from analysis. Heat maps of proteins quantitated in all nine samples were generated in Perseus, and settings of FDR < 0.05 and *s*0 = 0.1 were applied to generate volcano plots comparing the cell lines' proteomes. In analysis two, the set of proteins appearing in the MaxQuant output files of at least three different samples was evaluated, and summed *z*-scores (for each cell line's expression of that protein) with a difference of at least 1.0 were identified as being differentially expressed, visualized in heat maps generated in gplot.^82^ Enrichr^59–61^ was used to interpret analysis two, applying an adjusted *p*-value < 0.05 and drawing from the KEGG 2021 human database. Some of the Enrichr data (*P*-value < 0.05) was visualized using REVIGO^83^ and Cytoscape.^84^

Three replicate sub-samples were prepared from each of the human tissue samples CP565563, CP531533, and CP629057, and the nine sub-samples were treated and analyzed identically to the cell lysate samples (above).

## Data availability

All data from the cell lysate and human tissue sample experiments were deposited in the ProteomeXchange Consortium (https://proteomecentral.proteomexchange.org) *via* the PRIDE partner repository^85^ with identifier PXD036982.

## Author contributions

J. P., C. C., and A. R. W. conceived the concept of All-in-One DMF for miniaturized sample analysis. J. P., Shuailong Z., and M. E. O. fabricated the DMF devices. M. E. O. fabricated the manifold. A. A. S., V. R., and J. P. performed the surfactant optimization experiments on DMF. J. P. and C. C. performed the proteomic experiments on DMF. J. P., C. C., Shen Z., Y. H., E. Y. S., and H. P. carried out the proteomics analysis. B. B. L. and M. D. C. performed the cell culture experiments. J. P., C. C., Shuailong Z., E. Y. S., and A. R. W. wrote and edited the manuscript. All authors discussed the results and commented on the manuscript.

## Conflicts of interest

There are no conflicts to declare.

## Supplementary Material

SC-014-D3SC00560G-s001

SC-014-D3SC00560G-s002

SC-014-D3SC00560G-s003
