# Supplementary material for: All-in-One digital microfluidics pipeline for proteomic sample preparation and analysis
Source: Chem Sci. 2023 Feb 22;14(11):2887–900. doi: 10.1039/d3sc00560g (PMC10016607; doi:10.1039/d3sc00560g)
Supplement: SC-014-D3SC00560G-s002 [file SC-014-D3SC00560G-s002.pdf]

Electronic Supplementary Material (ESI) for Chemical Science.
This journal is © The Royal Society of Chemistry 2023

Supporting Information

All-in-one Digital Microfluidics Pipeline for Proteomic Sample Preparation and Analysis

Jiaxi Peng[1,2,3], Calvin Chan[1], Shuailong Zhang[1,6,7], Alexandros A. Sklavounos[1,2], Maxwell E. Olson[1], Erica Y. Scott[1,2,3], Yechen Hu[1,2,3], Vigneshwar Rajesh[1], Bingyu B. Li[1,2], M. Dean Chamberlain[1,2,3,8], Shen Zhang[4,9], Hui Peng[1,5], Aaron R. Wheeler[1,2,3*]

1. Department of Chemistry, University of Toronto, 80 St. George Street, Toronto, ON, M5S 3H6, Canada.
2. Donnelly Centre for Cellular and Biomolecular Research, University of Toronto, 160 College Street, Toronto, ON, M5S 3E1, Canada.
3. Institute of Biomedical Engineering, University of Toronto, 164 College Street, Toronto, ON, M5S 3G9, Canada.
4. Lunenfeld-Tanenbaum Research Institute, Mount Sinai Hospital, 600 University Avenue, Toronto, ON, M5G 1X5, Canada.
5. School of Environment, University of Toronto, 33 Willcocks Street, Toronto, ON, M5S 3E8, Canada.
6. School of Mechatronical Engineering, Beijing Institute of Technology, Beijing, 100081, China
7. Beijing Advanced Innovation Center for Intelligent Robots and Systems, Beijing Institute of Technology, Beijing, 100081, China
8. Saskatchewan Cancer Agency, University of Saskatchewan, 107 Wiggins Road, Saskatoon, SK S7N 5E5
9. Clinical Research Center for Reproduction and Genetics in Hunan Province, Reproductive and Genetic Hospital of CITIC-XIANGYA, Changsha, Hunan, 410000, China

[*]Contact details for the corresponding author: Aaron R. Wheeler

Email: aaron.wheeler@utoronto.ca; Tel: 416-946-3866; Fax: 416-946-3865

Address: 80 St. George Street, Department of Chemistry, University of Toronto, Toronto, ON, M5S 3H6, Canada

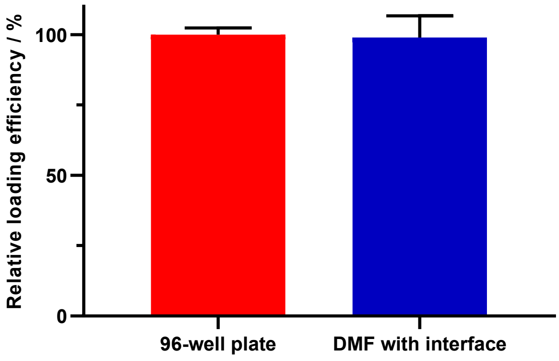

Fig. S1 Sample loading efficiency from conical bottomed 96-well plates (red) and the custom HPLC-DMF interface (blue). Identical volumes and concentrations of BSA digest standard (5 μL,1 ng/μL) were injected into the autosampler and analyzed by HPLC-MS. The average value of MaxQuant protein intensities for BSA for both systems were normalized to those from well-plates. Error bars represent 1 st. dev. from n = 3 replicates per condition.

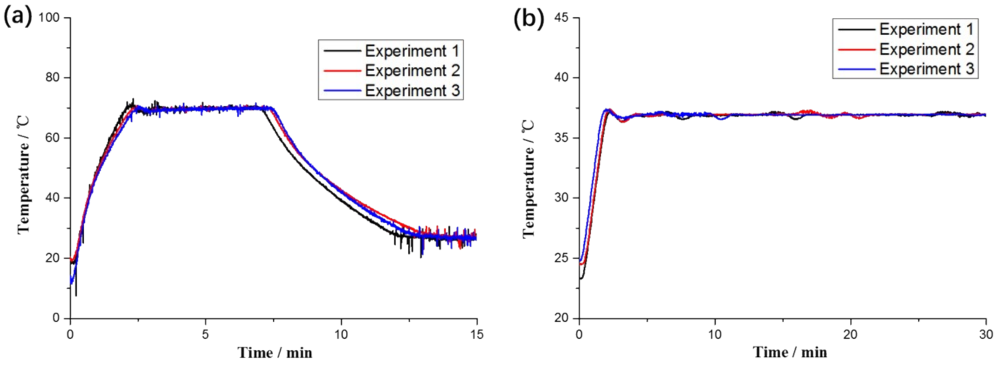

Fig. S2 Temperature control profiles of the two heating units. The temperatures in three successive runs (black, red, blue) were monitored by embedded thermistors during (a) a denaturation/reduction step (step 2) using the auxiliary heating unit, and (b) an enzymatic digestion step (step 4) using the integrated heating unit.

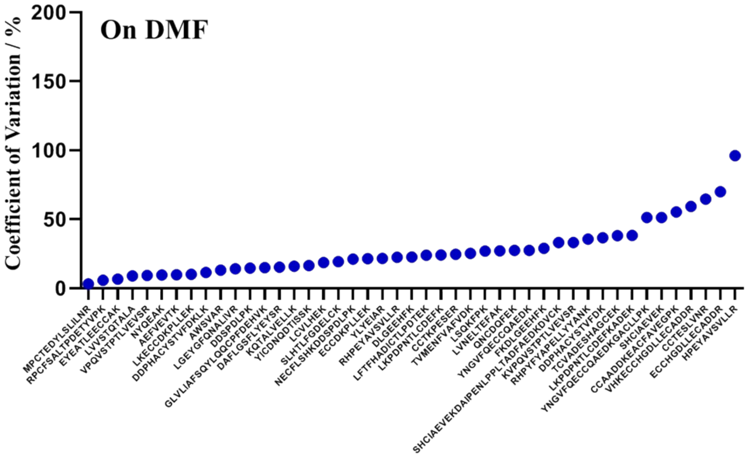

Fig. S3 Coefficient of variation (CV) of quantified peptides from BSA prepared on DMF using the All-in-One pipeline. Each blue spot represents the CV observed between the intensities of each peptide tagged with the light, medium, or heavy label.

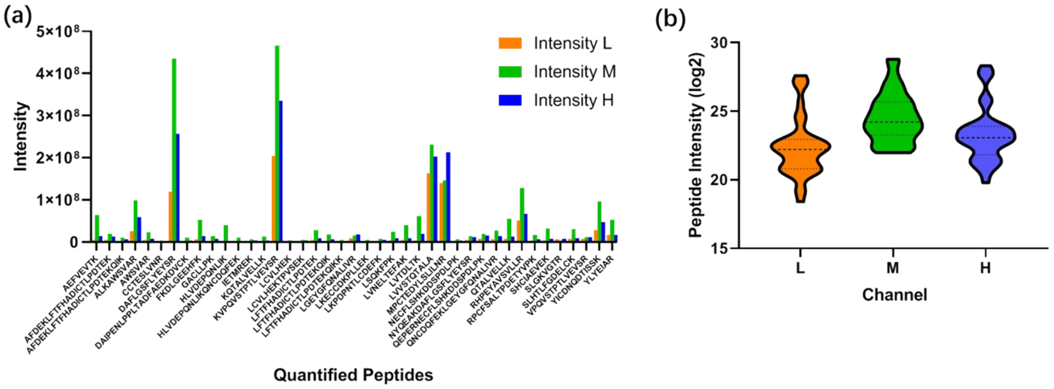

Fig. S4 Dimethyl labeling of BSA digests in tubes. (a) Intensity distributions of quantified peptides with light (L, orange), medium (M, green) and heavy (H, blue) label. (b) Violin plot of distribution of quantified peptide intensity (log2) from light (L, orange), medium (M, green) and heavy (H, blue) labeled channels for each of the 41 quantified peptides.

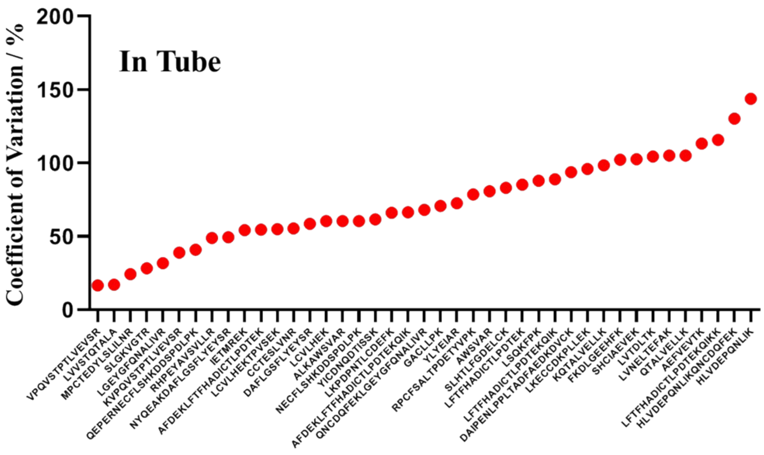

Fig. S5 Coefficient of variation (CV) of quantified peptides from BSA prepared in tubes. Each red spot represents the CV observed between the intensities of each peptide tagged with light, medium, or heavy label.

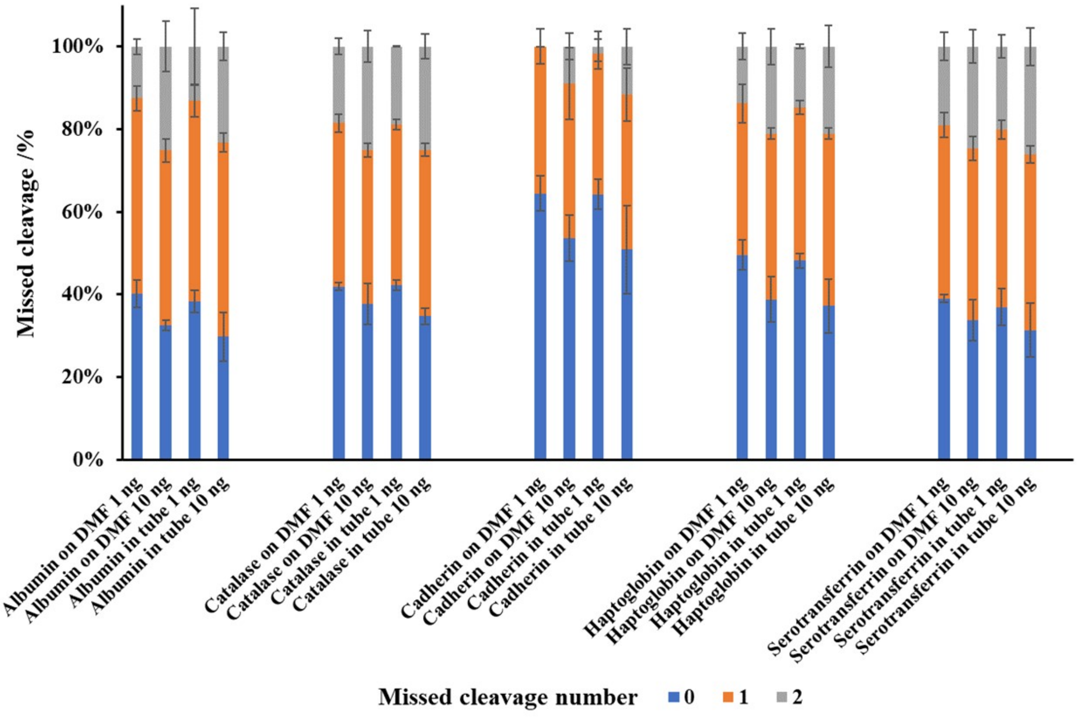

Fig. S6 Distribution of missed cleavages of identified peptides from a mixture of 1 ng or 10 ng of each 5 standard proteins (albumin, catalase, cadherin, haptoglobin, and serotransferrin) prepared on DMF and in tube. The number 0 (blue), 1 (orange), and 2 (grey) represents the number of missed cleavages on the peptide. Error bars are ± 1 st. dev. for n = 3 replicates per condition.

Table S1 Differentially quantified proteins between small breast cancer tumour sample (CP531533) and healthy breast tissue sample (CP565563). A positive log_2 fold-change represents higher quantitation in CP531533, while a negative log_2 fold-change represents lower quantitation in CP531533.

Entry name	Difference (log2 fold change)	-Log (P-value)
PGS2	1.5097	4.6449
ARY1	1.3308	2.1600
K1C18	1.1173	3.7753
UGDH	1.0673	2.4559
A0A0C4DGM1	1.0648	2.4298
ASPN	1.0617	3.0345
CYB5	1.0615	1.6568
ZA2G	1.0133	3.1131
IGLC7	1.0034	3.8842
S10AG	1.0018	2.9173
LUM	0.9148	3.0536
A0A0U1RQF0	0.8760	1.9864
COEA1	0.8603	4.5370
PRELP	0.7416	2.2750
IGHA1	0.7230	2.7757
CRIP1	0.7221	1.9329
TSTD1	0.6389	2.8417
FETUA	0.5086	2.3757
PDIA3	-0.4048	4.3970
HPT	-0.5067	3.0001
AMPL	-0.5585	3.1086
RRBP1	-0.5964	2.3236
COTL1	-0.6370	2.3605
PDIA4	-0.6595	2.2459
B2MG	-0.6860	1.9973
CALR	-0.6996	3.0303
PLSL	-0.7038	2.2539
G8JLJ2	-0.8113	2.2459
1A68	-0.8126	1.8990
COR1A	-0.9126	2.3672
D6RGG3	-1.0425	1.9797

Table S2 Differential proteins between large breast cancer tumour sample (CP629057) and healthy breast tissue sample (CP565563). A positive log_2 fold-change represents higher quantitation in CP629057, while a negative log_2 fold-change represents lower quantitation in CP629057.

Entry name	Difference (log2 fold change)	-Log (P-value)
PGS2	1.9054	5.3660
APOD	1.4028	2.8374
ASPN	1.2213	2.1361
A0A087X0Q4	1.1844	2.5353
LUM	1.0694	2.7730
PGS1	1.0584	2.7877
COEA1	1.0255	4.1710
KV401	1.0200	2.6614
AACT	0.9721	1.7297
A1AG1	0.9225	2.2135
PEDF	0.8875	2.3861
CO6A1	0.8784	4.8144
PRELP	0.8405	3.8573
SAA4	0.8358	2.4730
A0A075B7D0	0.8068	2.7062
VTNC	0.7548	3.1457
CO1A1	0.7428	2.0208
UGDH	0.7095	2.3879
CO6A2	0.7024	2.3957
HV372	0.6500	2.4502
IGHM	0.6445	3.4325
Q5VY30	0.6386	2.9890
E7ENL6	0.6227	3.4989
FINC	0.6195	3.0638
KV311	0.5986	2.5007
Q86W61	0.5837	2.1772
CFAB	0.5165	2.3721
A0A1B0GW44	0.5074	3.9852
CERU	0.5070	3.4725
A0A024R6I7	0.4957	2.4725
A0A2Q2TTZ9	0.4664	2.2429
CLUS	0.4394	2.3235
KV315	0.4329	2.5531
RL4	-0.3769	2.7780
PDIA3	-0.4229	3.5234
PRDX1	-0.5114	2.5553
AMPL	-0.5237	3.0538
B2MG	-0.5299	1.9586
CAPG	-0.5454	2.0225
H2B1L	-0.5563	3.5192
H10	-0.5588	2.3467
H4	-0.5728	2.3498
D6RGG3	-0.5757	1.8373

PLSL	-0.5794	1.8269
COR1A	-0.6014	1.9179
H7C3Z9	-0.6613	1.9516
TXND5	-0.6686	2.0276
CALR	-0.6798	2.7877
MYH9	-0.6997	1.7109
1A68	-0.7220	1.8237
FABP4	-0.8637	1.6655
PDIA4	-0.8780	2.4312
GPDA	-0.9933	1.6897
H14	-1.1660	1.8873
H15	-1.1741	2.1172
GSTM1	-1.6072	1.9245

Table S3 Differential proteins between small breast cancer tumour sample (CP531533) and large breast cancer tumour sample (CP629057). A positive $\log_2$ fold change represents higher quantitation in CP531533, while a negative $\log_2$ fold-change represents lower quantitation in CP531533.

Entry name	Difference (log2 fold change)	-Log (P-value)
ARY1	1.4919	1.7584
S10A7	1.1148	1.9707
K1C18	1.0970	3.2983
A0A1B0GTP7	0.8750	1.9208
ZA2G	0.6807	3.1148
GPDA	0.6371	2.1100
LG3BP	0.5929	2.3996
IGLC7	0.5393	2.5103
HBA	0.5240	2.1995
A0A0U1RQF0	0.4963	2.2113
IGHA1	0.4561	2.3325
PGS2	-0.3957	3.1020
KV315	-0.4488	2.6724
A0A1B0GW44	-0.4589	3.7245
PGS1	-0.4996	2.3232
K1C19	-0.5149	2.1819
CO6A1	-0.5190	3.5702
KV320	-0.5491	2.3448
S4R460	-0.5560	2.5544
A0A087X0Q4	-0.6037	2.0149
A0A075B7D0	-0.6398	2.9110
Q86W61	-0.6604	3.0031
SAA4	-0.6925	2.9318
KV401	-0.7706	4.3283
IGLL5	-0.8840	1.7762
AACT	-1.1247	2.5351
APOD	-1.1963	2.6265

Supplementary Design File

STL File 1. Design file for the DMF-HPLC manifold.

Supplementary Movie

Movie S1. Droplets with different DDM concentrations moving at different velocities.
